# Action Model for Unforeseen Inadequacy in Nurse Qualifications and Staffing: A Descriptive Study of the Experiences of Nurses and Nurse Managers

**DOI:** 10.1155/jonm/2346632

**Published:** 2026-06-22

**Authors:** Katja Lehtomäki, Kati Kannisto, Minna Stolt

**Affiliations:** ^1^ Department of Nursing Science, University of Turku, Turku, Finland, utu.fi; ^2^ Wellbeing Services County of Satakunta, Pori, Finland

## Abstract

**Aim:**

This study aimed to explore the experiences of nurses and nurse managers when using the Unforeseen Situations of Staffing and Qualifications action Model (USSQM) and to describe their views on the further development needs of the USSQM.

**Methods:**

A descriptive qualitative study was employed. Nineteen (*n* = 19) nurses and nurse managers from one department of a University Hospital in Finland were selected for semistructured focus‐group interviews by purposive sampling. Data were collected from January to October 2025; the interview guide focused on participant’s experiences of the use of USSQM. Data were analyzed using inductive content analysis.

**Results:**

The analysis of the results revealed six themes: (1) Integration of the USSQM into clinical routine, (2) Providing decision‐making support and practice clarity, (3) Strengthening the fundamentals of the USSQM, (4) Safeguarding nurse well‐being and ethics, (5) Fostering communication and cooperation, (6) Utilization of digital solutions. Nurses and nurse managers described that USSQM was mainly used to assess workload status and manage patient flow. Participants described experiencing a tension between their strong professional ethics and the fundamental objectives of the USSQM. Consequently, they expressed that it was challenging to follow the reorganization and prioritization guidelines dictated by the model.

**Conclusions:**

The participants described the USSQM as a practical and usable action model for daily practice. However, future refinement of the USSQM must focus on integrating objective workload indicators to reduce subjectivity and utilizing digital solutions for automated data retrieval. Additionally, adequate information and support from organizational leadership should be ensured during the implementation process to facilitate the comprehensive use of the USSQM.

**Implications for Nursing Management:**

Nurse managers especially experienced that USSQM gave them support in decision‐making; therefore, nurse managers play an essential role in ensuring the implementation of the USSQM in the units. They serve as role models and support nurses in decision‐making when tasks need to be reorganized or prioritized during periods of high workload.

## 1. Introduction

Adequate qualifications and a satisfactory staffing ratio for nurses are fundamentally linked to critical health outcomes, directly dictating the safety and quality of patient care [1–3] and the overall safety of the nurse’s clinical work environment [4, 5]. When nursing units are staffed with an optimal balance of personnel, appropriate skills, and experience, hospitals observe improved patient stability in managing high‐acuity cases, while simultaneously reducing the risk of adverse events and mitigating negative nurse outcomes, such as burnout and intention to leave [1, 3, 6]. To achieve optimal staffing conditions, healthcare facilities increasingly rely on evidence‐based methodologies [7, 8]. Because patient intensity constantly fluctuates, dynamic multifactorial approaches are now recognized as significantly better than simple, static patient‐to‐nurse ratios [7, 9, 10]. Despite the profound importance of achieving this optimal balance, determining the right staffing model remains a high‐stakes challenge. Consequently, frontline nurses frequently encounter situations characterized by high‐workload situations—often exacerbated by staff absences and intensive patient care needs [2, 5].

During the last few decades, both the worldwide shortage of educated nursing professionals and reduced financial resources have restricted the baseline staffing level (scheduled) and also reduced the skill mix, which no longer matches patient acuity [1, 2, 11–13]. Replacement of missing personnel or finding qualified nurses took a significant part of the nurse manager’s worktime; nevertheless, it is critical for safeguarding adequate staffing ratios. Utilization of a floating pool of nurses has been shown to be an effective and cost‐effective strategy for staffing shortfalls [14, 15]. However, a floating pool requires an initial financial investment [13] and not all healthcare organizations have such capacity or even willingness to do so. Despite all efforts, the difficulties caused by the nonexistence floating pools, the lack of trained nursing staff, and the strict criteria for using nursing agencies can become insurmountable challenges. Frequently, even one unforeseen absence and unexpected increase in the workload (e.g. due to higher patient acuity) can alter the staffing ratio from adequate to inadequate. The imperative for maintaining these ratios is fundamentally rooted in patient safety; low nurse‐to‐patient ratios directly compromise the delivery of safe care, increasing the risk of adverse events and missed nursing care. In these demanding environments, nurses must autonomously assess the workload situation and make critical decisions regarding which nursing tasks to prioritize [11–14, 16].

In the act of prioritization, nurses have to choose between patients, tasks, and duties and make a decision on who can wait, which task can be delayed, or even reducing the quality of care [13, 16–19]. Nurses are expected to apply critical thinking [19] when managing large amounts of information and when faced with complex and often emergent nursing situations. Moreover, their decision‐making is influenced by their previous education, clinical experiences, working environment, habits, values, attitudes, and beliefs [17–20]. Overall, the competency of nurses to make decisions regarding prioritization varies [21]. Prioritizing certain nursing tasks increases the risk of negative patient outcomes [1, 11, 22], but prioritization also has an effect on nurses’ outcomes. Prioritizing care forces nurses to make difficult decisions that often conflict with their professional values. Consequently, this discrepancy between ideal and actual practice drives ethical stress, job dissatisfaction, and staff turnover [17, 18, 20, 22, 23].

Addressing fluctuating workload demands requires the adoption of diverse and, at times, innovative strategies for maintaining equilibrium, while simultaneously navigating decision‐making processes that may conflict with personal or organizational values [24]. Griffiths et al. [6] provided a comprehensive review and summary of the literature about different tools and methodologies for assessing nursing workloads and determining adequate staffing. In addition, there are multiple tools for prioritization, mostly based on patient’s acuity, the hierarchy of needs, and time management [25, 26]. However, recent qualitative research emphasizes that these standardized measurements often fail to reflect the true complexity of nursing work. For instance, Benjamin et al. [27] highlight that frontline nurses frequently perceive a disconnect between objective workload metrics and the subjective, fluctuating intensity of patient care. Furthermore, exploring the practical application of national staffing tools, Dall′Ora et al. [28] found that nurses and managers may circumvent tools that do not align with their clinical judgment or local realities. From a leadership perspective, nurse managers face mounting challenges in balancing mandatory staffing policies with dynamic clinical needs, underscoring the need for tools that offer genuine decision‐making support rather than mere administrative tracking [29]. Consequently, no studies were identified that specifically addressed the development or evaluation of an action model designed to support the assessment of adequate staffing and to guide decision‐making in high‐workload contexts; this is particularly needed in situations where the reorganization or prioritization of nursing tasks is required.

In this study, we focused on an action model which has been developed to guide nurses and nurse managers in the assessment of a workload status in unforeseen inadequacy of staffing. This model is called the Unforeseen Staffing and Qualifications action Model (USSQM). The USSQM defines unit‐specific critical nurse staffing levels and qualification requirements (such as RN’s intravenous administration permits) utilizing a traffic‐light color‐coding system. In the USSQM, green indicates adequate staffing and sufficient staff or qualifications; yellow denotes minor observed deviations in staffing or qualifications; orange signifies a substantial deficit in staffing or qualifications; and red represents a critical shortage in staffing or competency. On the inpatient wards where the USSQM was piloted, the assessment of the workload status was based on an adequate nurse staffing ratio (volume‐based) and two patient‐related markers: the number of isolated patients and a subjective assessment of the care acuity of the patients. The action model guides the prioritization and reorganization of nursing duties during situations where deviations reach the orange or red levels. The model outlines critical tasks that must be prioritized, as well as noncritical tasks that can be safely postponed to the following shift or the next day. On the piloting wards, for example, medication administration and ensuring patient nutrition were identified as critical tasks. In contrast, rehabilitation activities and assisting patients with showering were considered less critical. Furthermore, the model provides guidance on implementing other supportive operational adjustments, such as modifying the execution of physician ward rounds and reorganizing team responsibilities. The fundamental objective of the model is to equip staff with clear guidelines for operating under conditions of inadequate staffing. Additionally, it serves as a decision‐making tool for assessing the workload level and determining how to prioritize activities during these exceptional situations. This qualitative descriptive study aimed to explore the experiences of nurses and nurse managers when using USSQM and to describe their views on the further development needs of USSQM.

## 2. Methods

### 2.1. Study Design

This study employed a qualitative descriptive study design to explore the experiences of nurses and nurse managers when using the USSQM and to describe their views on its further development needs. This approach provided the possibility to stay close to participants and describe their experiences and perceptions of a topic about which very little is known [30, 31]. The research was reported using the standardized criteria for reporting qualitative research (COREQ) [32].

### 2.2. Sampling and Recruitment

This study employed purposive sampling in a Finnish University hospital where the USSQM has been in use since 2024. Potential participants were eligible if they were working in the unit where the USSQM was implemented; thus, they had experience of its use. Medical professionals were excluded from this study because the study focused on the field of nursing sciences and was interested in the views of nurses and nurse managers on the USSQM [31, 33]. To ensure adequate prior informing, eligible participants received detailed information about the study from the named contact persons in each department in the selected University Hospital. Those willing to participate informed the contact persons, who then provided the researcher with their contact details or the participants contacted the researcher directly via email. Participants were required to provide written informed consent before taking part in the interviews. No personal data were processed during the interview sessions and during the transcription of the audio recordings. After the recruitment was completed, the researcher formed the participants into groups and arranged the time and place for the interviews; there were 19 participants who took part in 5 focus‐group interviews.

### 2.3. Data Collection

The data were collected using semistructured focus‐group (3–5 participants) interviews. The interviews were conducted face‐to‐face (4 out of 5) or remotely via video conferencing software (Microsoft Teams) (1 out of 5) depending on the participant group’s preferences by the same researcher. The first focus group served as a pilot group to test the interview guide, and no changes were made to it. This interview was included in the final analysis. In cases where video conferencing was used, the participants were co‐located in the same room while the researcher joined remotely to ensure consistency across data collection modes. This setup preserved the natural group dynamics and nonverbal communication between participants. To bridge the researcher’s remote presence, video was maintained throughout, ensuring the researcher could continuously observe all participants′ reactions and facial expressions. In a semistructured interview, all interviewees were asked the same questions, but follow‐up questions were allowed based on the interviewee’s answers and space provided for the interviewee’s individual opinions, experiences, and perceptions of the topic under study [34, 35]. The interview guide was developed for this study based on previous studies (e.g. References [13, 16, 36]).

The interview guide consisted of 12 open‐ended semistructured questions. The questions focused on the participants’ experiences of the use of USSQM, their responsibilities regarding the use of USSQM, their application of USSQM in daily work, and any promoting or hindering elements to the use of USSQM. As background information, the following information was collected: participants’ profession (RN, PN, nurse manager, other), years of experience in health care, years of experience in their current hospital ward, and the year in which the unit implemented USSQM. Data collection occurred between January and October 2025. The interviews were held in private meeting rooms and lasted 37–60 min. The researcher recorded the interviews with the participant’s permission and made notes during the interviews. The audio‐records were stored in the research organization’s secure cloud storage, with access strictly restricted to the research team.

### 2.4. Data Analysis

Inductive content analysis was employed focusing on manifest content because there is no previous research on the use of USSQM [37]. First, the audio recordings were transcribed verbatim with a secure speech‐recognition tool. The transcribed text was read while listening to the audio recording multiple times. Necessary corrections were made to the transcription. The transcribed data resulted in 60 A4 pages (Times New Roman, size 12, spacing 1.0). The unit of analysis was sentences or phrases containing words relating to experiences of the use of USSQM. These meaning units addressed specific research questions on the following: how nurses and nurse managers describe experiences of the use of USSQM; how nurses and nurse managers describe the usability of USSQM; what elements promote the use of USSQM or caused barriers to use; what are the further development needs of USSQM; and finally, how nurses and nurse managers describe the effects of the use of USSQM on experienced ethical stress. The meaning units were condensed, abstracted, and labeled with descriptive codes. During the inductive data analysis process, 171 codes were developed, and these codes were grouped into subthemes based on the similarities and differences. The subthemes were compared, and those with similar content were grouped into the same major theme [38]. In this study, data were determined through an operational method, that is, quantifying the number of new codes per interview [39]. During the analysis of the fourth interview, only a few new codes were identified, and following the fifth and final interview, no new data meaning units emerged.

### 2.5. Rigor and Trustworthiness

A four‐dimensional criterion comprising credibility, dependability, confirmability, and transferability was followed to ensure the rigor and trustworthiness of this study [39, 40]. Credibility was ensured by pilot testing the interview guide with one focus‐group, purposive sampling, and ensuring participants had the freedom to provide rich information. The data were participant‐driven until saturation was reached, followed by accurate transcription and data‐driven coding. Dependability was supported by maintaining a consistent methodological approach throughout the study. While the delivery mode varied between face‐to‐face and video conferencing, the same researcher conducted all interviews using a standardized semistructured guide to ensure uniformity in facilitation and probing. To ensure the accuracy of the data, all interviews were audio‐recorded and transcribed verbatim. The transcriptions were then cross‐checked against the original recordings to ensure technical and linguistic precision before analysis. The confirmability of the study was ensured by keeping detailed field notes and through the researcher’s reflexive diary notes during data analysis. This helped to ensure that the findings reflected the participants’ responses rather than the researcher’s own interpretations, and illustrative quotations were included when reporting the study findings. To ensure transferability, detailed descriptions of the study context, sampling strategy, participant characteristics, data collection, and data analysis processes have been provided.

### 2.6. Ethical Considerations

The study has been reviewed and approved by the Ethics Committee for Human Sciences Health Care Division at the selected University (No. TY/880/06.01.01/2024, 7 October 2024). Permission to conduct the study was obtained from this organization in November 2024. All participants were informed of the study procedure, purpose of the study, and anonymity and confidentiality of data and that they had the right to refrain from participation at any time without any consequences. Written informed consent was obtained from each participant prior to the interview. In this study, no participants declined or withdrew from the interview.

## 3. Findings

### 3.1. Characteristics of the Participants

A total of 19 nurses and nurse managers from five hospital wards participated in the study. The majority (*n* = 15) worked in wards where the USSQM was introduced in 2024, and the remainder (*n* = 4) in 2025. The majority of participants were nurses (registered nurses = 8, practical nurses = 5) and were experienced nursing professionals with more than 6 years of work experience (*n* = 16). However, the majority (*n* = 12) had less than 5 years of experience working in their current ward. Table 1 shows the characteristics of the participants.

**TABLE 1:** Participant characteristics.

Participant	Position	Age	Experience in nursing	Experience in current ward	USSQM introduced
1	Nurse manager	40–49	Over 16 years	Over 16 years	2024
2	Nurse manager	30–39	6–10 years	0–2 years	2024
3	RN	30–39	Over 16 years	0–2 years	2024
4	PN	Over 60	Over 16 years	Over 16 years	2024
5	PN	30–39	6–10 years	6–10 years	2024
6	Nurse manager	50–59	Over 16 years	3–5 years	2025
7	RN	30–39	11–15 years	6–10 years	2025
8	RN	Over 60	Over 16 years	Over 16 years	2025
9	PN	20–29	3–5 years	0–2 years	2025
10	Nurse manager	40–49	Over 16 years	Over 16 years	2024
11	RN	18–29	6–10 years	3–5 years	2024
12	PN	18–29	0–2 years	0–2 years	2024
13	PN	50–59	11–15 years	0–2 years	2024
14	RN	18–29	6–10 years	0–2 years	2024
15	Nurse manager	30–39	11–15 years	11–15 years	2024
16	RN	18–29	6–10 years	3–5 years	2024
17	Nurse manager	18–29	6–10 years	0–2 years	2024
18	RN	50–59	Over 16 years	0–2 years	2024
19	RN	30–39	3–5 years	0–2 years	2024

### 3.2. Codes, Subthemes, and Themes

The description of the experience nurses and nurse managers regarding USSQM was categorized into 22 subthemes, resulting in a total of six themes. The subthemes and themes are presented in Table 2. The categories included: (1) Integration of the USSQM into clinical routine, (2) providing decision‐making support and practice clarity, (3) strengthening the fundamentals of the USSQM, (4) safeguarding nurse well‐being and ethics, (5) fostering communication and cooperation, and (6) utilization of digital solutions.

**TABLE 2:** Summary of subthemes and themes.

Subthemes	Themes
Assessment of the workload status	Integration of the USSQM into clinical routine
Reporting the workload status	
The management of incoming patients	

Support for decision‐making	Providing decision‐making support and practice clarity
Anticipating future dilemmas	
Clarifying unwritten practices	

Needs arising from daily work	Strengthening the fundamentals of the USSQM
Understanding the purpose of the USSQM	
Educational needs to support implementation	
Shared understanding and rules	
Clear instructions	

Work‐related stress	Safeguarding nurse well‐being and ethics
Overall workload	
Professional ethics of the nurses	
Respect for others	

Delegation of tasks to another occupational group	Fostering communication and cooperation
Multidisciplinary cooperation within the unit	
Cooperation with stakeholders	
Directors’ commitment to the use of the USSQM	
Shared and agreed rules secured the cooperation	

User‐friendly format	Utilization of digital solutions
Integration with other electronic systems	

#### 3.2.1. Integration of the USSQM Into Clinical Routine

Nurses and nurse managers described that USSQM had become a part of their daily work, and the USSQM was implemented in their daily work, but its use as a daily tool varied in terms of its comprehensiveness. Nurses and nurse managers mentioned that the workload status of the wards was assessed and reported on in accordance with the guidelines. In some wards, assessing the status was described as being the responsibility of everyone, but in some wards, the assessment was often left to a single named person. In these wards, the nurse in charge or nurse manager was named as the person responsible for assessing the status.

“It’s mainly the primary nurse (who assesses the situation)”

Assessment of the status was perceived to be difficult; in particular, there was mistrust about the ability to maintain objectivity and the influencing effects of previous experiences. However, participants felt that the greatest benefit was achieved when the assessment was conducted together and everyone in the shift was aware of the status.

“And I′m sure there will be a lot of discussion about it (status). If you feel unsure about making that decision, don′t hesitate to ask. We also have the wards divided into groups, so you can′t always know what the situation is in another group.”

In each focus group, participants described that the status was regularly reported through the agreed channel. The frequency of reporting varied between wards depending on how familiar the nursing personnel was with the assessment. Reporting the current status in the ward was described as important for making the existing workload status visible.

“There are more and more patients requiring intensive care in our wards. But then if there are patients who cannot be managed, and we are unable to treat those who are already in the ward. So how do we get the new ones treated? That′s why the USSQM is good.”

The management of incoming patients was considered an important added value of the USSQM. According to the guidelines, the number of new patients being admitted to the ward is limited when the USSQM status is orange or red. This was described as increasing job satisfaction and well‐being.

“So then there must be some way that we can perhaps limit the number of overflow patients during this shift. So, there is some way to avoid a situation where there is a staff shortage, but patients keep coming and coming.”

#### 3.2.2. Providing Decision‐Making Support and Practice Clarity

The USSQM functioned as a structural support system that clarified professional expectations and assisted in clinical prioritization. Nurse managers described that they had more experience of support in decision‐making than nurses. None of the nurse managers expressed confidence or comfort in making prioritization decisions and the USSQM was described as a decision‐making support tool, particularly in the assessment of adequate staffing levels and guiding nurses in the reorganization of their tasks, especially in new or infrequent situations.

“I′ve been able to explain why we should leave this job for now… And then I could say, hey, we have this situation now, maybe that made it easier for me to tell the staff to take it easy for a while.”

The participants described that the wards had unwritten guidelines for reorganization and prioritization, but the USSQM was considered a good tool, especially for new and nonexperienced nurses. By formalizing these previously tacit guidelines, the model offered a structured framework that could potentially reduce cognitive load and support more consistent prioritization practices.

“This is good in a situation where there is someone completely new here and these (unwritten guidelines) are strange.”

In some wards, where the USSQM had already been widely adopted, the model enabled a shift from reactive to proactive practice. In these wards, participants felt it was possible to foresee and prepare for potential dilemmas and organize tasks well in advance.

“My shift is normal, but I can see that there won′t be enough nurses tomorrow morning. So, then I shouldn′t make too many promises to the overflow patients the next night. When the next shift is already in trouble.”

#### 3.2.3. Strengthening the Fundamentals of the USSQM

Nurses and nurse managers described that understanding the fundamentals of the USSQM strengthens its use. They described how restricted nurse staffing ratios are affecting their daily work and even a single unforeseen absence can force a reorganization and prioritization of tasks and duties. One nurse manager believed that there would be less need for this kind of action model if there was more flexibility in the overall number of nurses allowed. However, the participants described that overflow patients were more common, as were high‐workload statuses, so action models were necessary.

“But now they have been talked about, or perhaps they have been on people’s minds more, because there have been so many cases of overflow situations. And then, as we’ve actually had patients beyond the official capacity, there has been a more practical need to go and check, like, oh right, there was that guideline—what did it say again?”

Different challenging situations and the need for guidance arise in daily work, and in one ward, these needs have led to a local update in the guiding sections of the USSQM.

“It has been reviewed from time to time at ward meetings when there has been a need for updates or some changes have been made to it. I would think that perhaps the ward assistant and I have discussed that update at this point…but it has been viewed together at ward meetings”

Understanding the purpose of USSQM as a whole was described as being challenging in the big picture. Participants believed that they have understood the purpose of the USSQM as a guiding tool that helps assess the status and provides guidance for reorganization and prioritization, but they assumed that not everyone understands this.

“I think it′s about understanding. How it serves employees, the entire work community, and external operations. Because it was something that people weren′t aware of. To be honest, even though I was a supervisor, I didn′t understand it at first, so it didn′t fully sink in for me either. Now it has become familiar to me.”

Participants described that not all staff members fully understood that USSQM was developed to help them in high‐workload situations and support them in decision‐making, not to blame them for unfinished tasks and duties. The use of the USSQM was primarily associated with unforeseen inadequacy caused by staff absenteeism, rather than during periods of high patient acuity. That is why, encouragement from nurse managers and leading by example were also considered important.

“It is also emphasized that this applies to other situations as well, not only to situations of absence.”

“This is always emphasized that there is the prioritization model. That we act according to it if we cannot manage everything.”

Education and sharing information were described as playing a fundamental part in a successful implementation, as well as allowing time for employees to become familiar with the use of the USSQM. Participants described that, in a state of continuous changes, time should be allowed for the implementation, and the use and purpose of the USSQM should be reviewed regularly.

“I think it should be done at regular intervals… I think it should be done for many years, like a constant reminder in the field, so that it becomes a way of doing things.”

The participants considered it important that all users in the same hospital department have the same understanding and rules for using the USSQM. The assessment of workload status was seen as critical, and the participants argued that the USSQM should be so clear that it minimizes the possibility of subjective interpretation. A clearer definition of the difference between statuses, particularly between the yellow and orange statuses, was deemed necessary.

“When it comes to intensive care and high workload, it′s such a subjective experience. And the more stressed the nurse is, the more stressful the situations feel.”

“It may have been a little confusing, because when you see that orange color, you might think that there are a lot of isolated patients there, patients requiring intensive care. But there is no limit to how many there are, so it′s kind of unclear where the line is drawn.”

The majority of participants felt that the USSQM’s instructions for reorganizing and prioritizing nursing tasks and duties were comprehensive.

“I watched this myself at the time, these critical actions. I couldn′t have left anything out.”

#### 3.2.4. Safeguarding Nurse Well‐Being and Ethics

Nurses and nurse managers experienced that the use of USSQM has made an impact on employees’ well‐being at work and the effects were seen as multifold. At the beginning of implementation, nurse and nurse managers described how USSQM had caused stress to employees. They assumed that the lack of knowledge and understanding of the fundamentals of USSQM, as well as a general resistance to change was the reason for the caution. However, they also described the stress had eased as the employees gained more experience with USSQM and its use in daily routines.

“And that′s what we heard from the field at first. These traffic lights (assessing the status) caused stress for everyone. The thing was that we have very precise nursing colleagues here, and they want to do everything as well as possible. The USSQM caused stress at first because it increased workload.”

The poor overall situation with patient flow and the increased need for care were described as the main reason for experiencing stress, and USSQM was not seen as a solution to this situation.

“A general feeling of being inadequate. This can create even more stress. You want to give even more, but you don′t have the opportunity.”

Nurse managers described experiencing ethical stress when balancing decision on whether to follow strict financial regulations or support employees’ well‐being in the situation where an extra‐employee was needed.

“Taking on extra resources—well, that′s not something you want to do. You always have to think about the savings side, that there are no extra resources available and that, in principle, you shouldn′t use them. Nothing other than very carefully consideration, as has been done.”

“And then, employees are tired and the workload is heavy, but then there is the situation where you have to take the overflow patient or do something else. So, you sometimes wonder what kind of ethical practice is part of my job as a nurse manager.”

Following the prioritization guidelines of the USSQM was sometimes seen as conflicting with the professional ethics of nurses and their desire to do their job well, causing ethical stress. However, some participants saw the potential of USSQM to relieve the ethical stress of nurses in prioritizing tasks and duties if they were prepared to compromise their values and follow USSQM’s guidelines.

“It′s not necessarily the nurses′ fault in that situation (high workload). I check the instructions on the USSQM, and that′s the only thing that reminds me that I have permission to act like this when necessary. That there are specific instructions for that. But it′s really difficult.”

Nurses described some unpleasant situations and feelings of guilt when nurses have prioritized their tasks in heavy‐workload situations and other members of the work community (e.g., medics and physiotherapists) have criticized them for not doing their tasks. To avoid conflicts and guilt, it is important that everyone is aware of the workload situation and also respects agreed guidelines of the USSQM.

“It′s annoying when a nurse goes through that ethical dilemma and leaves something undone by following the instructions. Then someone immediately asks why it hasn′t been done.”

“Then you get that feeling as a nurse that you haven′t done your job.”

Nurse managers described how nurses had contacted them outside of working hours. Nurse managers considered that if the USSQM was implemented well and employees followed its guidelines, it would decrease the need to disturb managers during their free time.

“Let′s raise awareness about it. It might give me a little peace and quiet on the weekends, too.”

#### 3.2.5. Fostering Communication and Cooperation

Communication and cooperation among the stakeholders associated with the use of USSQM were described an essential part of its use. Nurses and nurse managers, especially named medics, therapists (physio and occupational), environmental service workers, and ward secretary as their primarily cooperators, and communication with them was described essential, but easily forced into the background during a high‐workload status. Some of the participants described how they have effectively delegated tasks to the ward secretary during a high‐workload status. Although they recognized the potential for cooperation with therapists, there is still no systematic way in place to communicate to therapists the high‐workload status of a ward.

“The secretary works in the office and is very aware of what is happening and the overall situation, and also provides support in various ways. In times of high workload, she can take on some additional tasks herself if time allows.”

“Communication is always challenging. There is certainly potential for improvement in making it more effective and in keeping everyone informed. In my role as the person conveying the information, I have discussed such situations with the physiotherapists. They have shown a positive attitude and a willingness to assist with patients’ toilet visits, and they have constructively considered how they can contribute in those situations.”

The participants viewed daily multidisciplinary meetings and whiteboard updates as potential first steps toward better communication. A short briefing about the situation ensures that everyone in the team knows that prioritization is needed and an information message on the white board avoids blame‐sharing caused by uncertainty.

“When I think about it, even a small morning briefing could help. We could go over who is where and what each person’s responsibilities are. And then, kind of like… okay, today is green—nothing urgent. But if it were red, we would go through it and say out loud, “Hey, this is the situation now,” and then resolve it quickly. We would prioritize tasks accordingly. That way, it would somehow get addressed, because I feel like it currently gets overlooked.”

“You can hear it straight from colleagues, or in the next shift: ‘Oh, that hasn’t been done. Well, why hasn’t it been done?’ So clearly, it may be necessary to inform them: ‘Hey, did you know that the previous shift was like this?’”

Cooperation with so‐called coordinating nurses was described as an essential part of the use of the USSQM. Coordinating nurses cooperate with the hospital departments and coordinate patient transition from one unit to another. Reporting USSQM’s status via agreed channels was described as a way to manage incoming patients, especially overflow patients. In this cooperation, the participants described the necessity of trust. Nurses and nurse managers experienced that, in some cases, the coordinating nurses have not trusted the ward’s assessment of the USSQM status and therefore pressured them to take in more patients. These experiences decreased the feeling of trust and weakened collaboration. That is why nurses and nurse managers emphasize the need for trust to enable effective collaboration in the best interest of the patients.

“At the beginning of the year, it was still somewhat questioned why you have the light status on… Thank goodness it didn’t continue, because even during the summer there were still those guilt‐inducing calls. Being on each other’s case like that doesn’t help, because it doesn’t relieve the pressure when everyone is under stress.”

The Department’s directors, such as the director of nursing and the medical director, were described as having an important role in fostering collaboration within the department and also with other stakeholders. Nurses and nurse managers were unaware of by what means and how often the directors monitored the ward’s reported USSQM status. During a period of a high workload, it was considered especially necessary that directors obtained sufficient understanding of the ward’s situation. This would enable them to provide relief by managing patient flow while the ward was solving the situation.

“So, the idea is that when staffing is normal, any available beds are used for overflow patients, no matter the situation. You have to come up with quite elaborate explanations if you don’t want to use the extra bed. And that’s what the staff doesn’t understand, because in the previous organization it was just a single phone call to the director: ‘We won’t use the overflow bed now’, whereas here it has to go through several layers of approval.”

One nurse manager noted that shared and agreed rules were considered essential for cooperation and collaboration, including assessment of workload status and managing overflow patients. At the same time, staff expressed the need for some flexibility.

“Even if some flexibility could be allowed, the rules still need to be maintained. As mentioned, the perception of workload is subjective, and without clear guidelines, one nurse could decide ‘I won’t admit any patients this evening’, simply based on personal preference. However, there should ideally be slightly more leeway than what the current guidelines allow.”

#### 3.2.6. Utilization of Digital Solutions

Nurses and nurse managers described that the USSQM, in its current visual form, was good in terms of usability. However, they assumed that by utilizing digital possibilities, the model could be made even more user‐friendly. Participants described how paper‐based instructions are easily lost, which can negatively affect their use in practice.

“Yes, and all those paper notes… there are, like, five million of them on the board, so they really just get lost.”

Several participants explained that a digital version of the action model, capable of collecting additional information through integration with other systems—such as the electronic patient record (EPR) system—could represent a potential future development direction for the USSQM. In particular, they noted that integration with the patient record system could provide real‐time data on intensity of care needed for a patient, thus supporting the workload assessment.

“If we could get the care intensity data through EPR, then we could develop this model further by integrating it there. That would be the ideal model.”

According to participants, adherence to prioritization guidelines might be easier if the USSQM operated on a digital platform that automatically generated context‐specific recommendations based on workload assessment data. Nurses and nurse managers also suggested that a digital USSQM could facilitate the storage of workload monitoring data, enabling the examination of potential associations between patient safety incidents and periods of high workload.

“Well, the thing is, they just get left somewhere, and later you’d have to go and fetch them. Like, if someone asks what kinds of situations have occurred—when there have been, for example, so many patients or very high care intensity—then they’re kind of just… somewhere.”

## 4. Discussion

The aim of this study was to explore the experiences of nurses and nurse managers when using the USSQM and to describe their views on the further development needs of USSQM. The USSQM was developed firstly to guide nurses and nurse managers in the assessment of workloads and the adequate qualifications of staff, and secondly to guide them in decision‐making as regards the need for reorganization and prioritization of nursing task and duties in inadequacy staffing situations. To ensure close alignment with the study’s purpose, these findings are discussed through the empirically derived themes of the participants′ experiences.

### 4.1. Integration of the USSQM Into Clinical Routine

Regarding the integration of the USSQM into clinical routine, the findings of this study demonstrated that the USSQM is usable and has become a part of nurses’ daily work. The implementation of the USSQM led to a structured assessment of the units’ workload, and its reporting was directed by the department directors, which had not been routinely conducted prior to its adoption. To maintain an adequate staffing ratio, the assessment of workload status was perceived as important to make the existing workload status more visible and enable informed decision regarding the incoming patient flow. The better management of patient flow was perceived to reduce workload. However, the results show that there were variations in the implementation and the comprehensive use of the reorganization and prioritization guiding parts of the USSQM. Nevertheless, nurses and nurse managers described the USSQM’s potential as a new approach to manage workloads and as a tool to support decision‐making on prioritization, and their willingness to continue the use of USSQM confirmed the need for this kind of action models [24].

### 4.2. Providing Decision‐Making Support and Practice Clarity

In terms of providing decision‐making support and practice clarity, the USSQM holds promise as a tool to guide prioritization, although the findings from this study reinforced the fact that making decision to prioritize is not easy [17]. In this study, nurse managers were more satisfied with the support the USSQM provided for decision‐making, especially in infrequent situations. Although following the prioritization guidelines was perceived as challenging, the guidelines of the USSQM’s were nevertheless considered to be comprehensive in terms of content. The ability to make decisions is shaped not only by clinical experiences, education, or competencies but also by resource constraints, institutional policies, and interprofessional dynamics and relationships [21].

### 4.3. Strengthening the Fundamentals of the USSQM

Despite its potential, participants highlighted the need for strengthening the fundamentals of the USSQM. Implementation of action models, or guidelines, is known to be complex in nursing care [38], and despite the potential benefits, successful adoption is often hindered by a variety of barriers [39]. According to this study, implementation variability was attributed to the lack of understanding of the fundamentals of USSQM, time pressure, educational needs of the users, cooperation in multidisciplinary level, and even attitudes and values. These identified barriers to comprehensive implementation of USSQM are related to findings from previous studies where implementation barriers were related to personal factors, guideline‐related factors, and external factors [38, 41]. The findings of this study are important considerations for the continued use of USSQM and its potential extension into broader use. Units currently employing the USSQM should be provided with regular continuing evaluation and education, as well as targeted support to address the challenges identified in this study. In future, a structured implementation plan [40] could improve adherence and comprehensive use of the USSQM.

### 4.4. Safeguarding Nurse Well‐Being and Ethics

A critical aspect of using the USSQM involves safeguarding nurse well‐being and ethics**.** Nurses described a conflict between making decision to prioritize and their own professional values. To make decisions, nurses obtain the necessary information and interpret it in order to make judgments and actionable decisions. The commitment of nurses to follow their ethical values and professional codes is high, despite the pressures of workloads, resource limitations, and the emotional burden of ethically complex situations [17, 42]. Despite positive experiences with the tool, the poor overall situation in healthcare with inadequate baseline staffing [13] and increased need for care [16] was identified as a reason why such action models are primarily needed. Strengthening the baseline nurse staffing or investment in a floating pool of nurses [13, 15, 16] could be a solution to ensure that the USSQM’s original purpose is fulfilled, so that prioritization does not become so evident and continuous in everyday work, consequently contributing to improved staff work well‐being.

### 4.5. Fostering Communication and Cooperation

To maximize the tool’s effectiveness, fostering communication and cooperation is essential. The findings indicated that multidisciplinary cooperation heavily influences the use of the USSQM. Through institutional policies especially, the organization could provide even stronger support for nurses in following the guidelines. Under the high‐workload status, nurse managers have sought to lead by example and have actively encouraged nurses to follow the prioritization guidelines. Findings from previous studies confirmed that open and preparatory discussions [17, 24], complemented by targeted training and coaching [26], and interprofessional collaboration [21, 43], could provide nurses with more robust support. In the context of prioritizing nursing tasks, traditional hierarchies between nurses and physicians should be set aside; therefore, it is particularly important to ensure that the decision‐making process is transparent with effective communication and teamwork [21]. Moreover, experiencing successful, collaborative decision‐making can mitigate ethical dilemmas for nurses.

### 4.6. Utilization of Digital Solutions

Finally, regarding suggestions for improvement, participants emphasized the utilization of digital solutions was seen as a future option. Obtaining up‐to‐date data from patient record systems and staff calculation could serve as indicators for assessing workload, directly addressing the critiques regarding subjective assessment. Digital solutions with automatized recommendations for reorganization and prioritization could also improve following the prioritization guidelines. The findings of this study suggest that integrating these digital elements is a necessary next step in the further development of the USSQM.

## 5. Limitations

Several limitations should be considered when interpreting the findings of this study. First, this research was conducted within a single hospital department, which may influence the transferability of the results to other clinical settings or geographic regions. The study was conducted in a single hospital department, where the reporting of workload statuses was guided, and prioritization practices were largely standardized across all wards using the model. The objective is not to generalize findings to a broad population, but rather to provide a deep, contextualized understanding of the experiences when using USSQM.

The sample size was guided by the principle of information power [44]. Given that the participants possessed specialized expertise in the use of the USSQM and the study’s aim was highly focused, a sample of 19 was deemed sufficient to achieve data saturation. However, as the USSQM is a novel tool with no direct counterparts in existing literature, these findings should be viewed as an initial, foundational exploration. Future multicenter research is required to evaluate the model’s utility and sustainability on a broader scale.

A methodological limitation of this study is the use of a mixed‐mode approach for focus‐group interviews, which were conducted both face‐to‐face and via video conferencing (Microsoft Teams). During the research process, we recognized that differences in the interview medium can influence group dynamics, rapport‐building, and response patterns; face‐to‐face interactions often facilitate more organic dialog through subtle nonverbal cues, whereas virtual settings can sometimes hinder spontaneous interaction due to technical constraints or reduced social presence. However, we anticipate that this mode effect was minimal, as only one of the five interviews was conducted via video conferencing (Microsoft Teams). Furthermore, in this single virtual instance, the participants were colocated in the same room while the researcher joined remotely. This setup successfully preserved the natural group dynamics and nonverbal communication between the participants. To bridge the researcher’s remote presence, video was maintained throughout, ensuring we could continuously observe all participants′ reactions and facial expressions.

Finally, to maintain the dependability of the data, the same researcher moderated all interviews using a standardized interview guide to ensure consistency in facilitation. Furthermore, group sizes were kept consistent (3–5 participants) across both modes to foster similar levels of engagement. All interviews were transcribed verbatim and verified for accuracy to ensure that the depth and quality of the data remained comparable regardless of the interview format.

## 6. Conclusions

This study provides valuable information on the newly developed action model, the USSQM, and the experiences of nurses and nurse managers as regards its use and their perspective on any further development. USSQM is a new approach to the assessment of workloads and a guide to reorganization and prioritization, the experiences of the use of the USSQM were positive, and there was confidence in its potential. However, additional research is needed; especially, the content of the USSQM should be examined further. The participants highlighted the need to include a multifactorial patient classification system in the USSQM, which would ensure a more objective way to assess the workload status and thus guarantee an adequate staffing level in high‐workload situations; in addition, they suggested the need to improve trust between all stakeholders. In the future, examination of the following factors should be considered: whether the guidelines regarding reorganization and prioritization are sufficient, the reasons why the threshold for following them is so high for nurses, and how nurses’ decision‐making in prioritization situations could be better supported. The focus should also be directed toward ensuring the implementation of the USSQM, and a structured implementation plan should be developed.

## Author Contributions

Katja Lehtomäki contributed to the conception and design of the study, data collection, data analysis, interpretation of the findings, and drafting of the manuscript. Kati Kannisto and Minna Stolt contributed to the conception and design of the study, developed the research protocol and proposal, and provided supervision and critical revision throughout the research process and manuscript preparation.

## Funding

This study was supported by the Tyks Foundation, which awarded a general working grant to Katja Lehtomäki.

## Conflicts of Interest

The authors declare no conflicts of interest.

## Data Availability

The data that support the findings of this study are available upon request from the corresponding author. The data are not publicly available due to privacy or ethical restrictions.
